# D-KEFS ST Failure Identifies Multiple Sclerosis Patients With Worse Objective and Self-Perceived Physical and Cognitive Disability

**DOI:** 10.3389/fpsyg.2019.00049

**Published:** 2019-01-24

**Authors:** Alice Riccardi, Marco Puthenparampil, Francesca Rinaldi, Mario Ermani, Paola Perini, Paolo Gallo

**Affiliations:** ^1^Multiple Sclerosis Centre, Department of Neurosciences DNS, Padova, Italy; ^2^Multiple Sclerosis Centre, University Hospital-Medical School, Padova, Italy; ^3^Department of Neurosciences, University Hospital-Medical School, Padova, Italy

**Keywords:** Multiple Sclerosis, neuropsychological assessment, executive functions, Delis-Kaplan Executive Function System Sorting Test, Brief Repeatable Battery of Neuropsychological Test

## Abstract

**Background and Objectives:** The Brief Repeatable Battery of Neuropsychological Test (BRB-NT) does not explore the executive functions. We combined BRB-NT and Delis-Kaplan Executive Function System Sorting Test (D-KEFS ST) to obtain a more comprehensive evaluation of cognitive impairment in Multiple Sclerosis (MS) patients.

**Methods:** 137 Relapsing Remitting MS (RRMS) patients underwent a detailed neuropsychological assessment including BRB-NT, D-KEFS ST and self-administrated questionnaires, namely the Multiple Sclerosis Neuropsychological Questionnaire (MSNQ), the Fatigue Severity Scale (FSS) and the Beck Depression Inventory-Second Edition (BDI-II).

**Results:** Fifty-four patients (39.4%) had normal scores in each item of both batteries (cognitive normal), while 64 patients (46.7%) failed in at least one test of BRB-NT but not of D-KEFS ST (BRB-NT impaired) and 18 (13.1%) failed in at least one test of both batteries (BRB-NT+D-KEFS ST impaired). Only one patient (0.7%) failed in D-KEFS ST, but not in BRB-NT and was excluded from further analysis. BRB-NT+D-KEFS ST impaired patients had a significant higher mean disease duration and median EDSS score (15.5 ± 13.6 years and 3.5, respectively) compared to those with only BRB-NT impaired (7.9 ± 9.2, *p* < 0.01 and 2.5, *p* < 0.05) and with cognitive normal patients (6.7 ± 9.4, *p* < 0.005 and 2.0, *p* < 0.01). SDMT was more frequently impaired in BRB-NT+D-KEFS ST impaired patients (77.8%) compared to only BRB-NT impaired ones (20.0%, *p* < 0.001). The failure in D-KEFS ST was associated with the number of failed BRB-NT items (OR 1.46, IC95% 1.07–1.99, *p* < 0.05) and with pathological SDMT *z*-value (OR 10.56, IC95% 2.50–44.66, *p* < 0.005). Compared to BRB-NT impaired patients and the cognitive normal ones, BRB-NT+D-KEFS ST impaired patients had significant higher MSNQ (*p* < 0.01) and BDI-II (*p* < 0.05) values.

**Conclusion:** D-KEFS ST did not increase the number of cognitively impaired MS patients identified by BRB-NT, but provided a more comprehensive evaluation of cognitive decline. D-KEFS ST identified a subgroup of patients with increased self-perception of cognitive decline, depression and higher physical disability.

## Introduction

Multiple Sclerosis (MS) is a chronic disabling neurological disorder affecting both the white and gray matter of the central nervous system. Up to 70% of MS patients develops various degrees of cognitive impairment that can be observed since early disease phases, sometimes at clinical onset (Chiaravalloti and DeLuca, 2008; Amato et al., 2010; Nourbakhsh et al., 2016).

Early cognitive impairment is considered a severe prognostic factor, tends to a progressive worsening and is not always linked to the accumulation of physical disability (Amato et al., 2006b; Moccia et al., 2016). Verbal and visuospatial memory, information processing speed and sustained attention are the most commonly compromised cognitive functions in MS. It is currently accepted that cognitively impaired patients are more likely to be unemployed or experiencing restrictions in social activities and household responsibilities (Rao et al., 1991a,b; Amato et al., 2001; Morrow et al., 2010; Ben Ari Shevil et al., 2014).

The most widely used tool to assess the cognitive profile in MS patients, the Brief Repeatable Battery of Neuropsychological Test (BRB-NT) (Rao, 1990), mainly explores memory, attention and information processing speed (with a sensitivity of 71% and a specificity of 94%), but does not properly investigate the executive functions. Indeed, about 17% of MS patients reveals failures in abstract and conceptual reasoning, fluency, planning and organization (Drew et al., 2008), thus limiting the ability of solving problems and making decisions.

To date, different tests have been applied to investigate various components of the executive domain in MS. Among these, (i) the Stroop Test (Stroop, 1935), that evaluates the ability to inhibit an automatic response while performing a task based on conflicting stimuli (Baysal Kıraç et al., 2014; Patti et al., 2015), (ii) the Tower of London (Shallice, 1982), that considers the ability to plan and to generate a sequence of goal-directed actions (Voelbel et al., 2011; Owens et al., 2013), and (iii) the Wisconsin Card Sorting Test (WCST) (Grant and Berg, 1948), that explores cognitive flexibility, problem solving and rule learning (van der Hiele et al., 2015; Koini et al., 2016).

Recently, the Delis-Kaplan Executive Function System (Delis et al., 2001) (D-KEFS) has been proposed for a more comprehensive evaluation of the executive functions. Indeed, D-KEFS has provided evidence for specific deficits in several neurological disorders, including traumatic brain injury, (Strong et al., 2011; Heled et al., 2012), Parkinson’s disease (McKinlay et al., 2010), dementia (Huey et al., 2009; Gansler et al., 2017), lateral prefrontal cortex lesions (Yochim et al., 2007), agenesis of the corpus callosum (Marco et al., 2012), stroke (Al-Dughmi et al., 2017), and amyotrophic lateral sclerosis (Libon et al., 2012). In particular, D-KEFS Sorting Test (D-KEFS ST), a subtest of this battery, was able to explore reasoning, categorization abilities, problem solving, abstraction, flexibility of thinking and concept-formation skills, providing a good validity (Parmenter et al., 2007) and an adequate reliability (Strauss et al., 2006). Despite WCST and D-KEFS ST gave comparable results in MS (Parmenter et al., 2007) relevant differences exist between these two tests. In particular, D-KEFS ST, but not WCST, discriminates the various components of the executive functions allowing the identification of specific impairments (Lezak, 2004). Moreover, D-KEFS ST investigates both verbal and non-verbal modalities of concept formation, does not employ the right/wrong feedback procedure (that could discouraged some individuals), and allows repeated measures, thus providing alternate forms. Recently, an abbreviate form of D-KEFS ST, i.e., the Card Sort 1, turned to be a good screening test for executive dysfunctions (Gromisch et al., 2016). Finally, an Italian study (Mattioli et al., 2014) provided normative data for D-KEFS ST. All these features might support D-KEFS ST as a compelling instrument to estimate an impairment of executive functions also in MS patients (Benedict et al., 2002).

However, it has to be pointed out that cognitive tests do not exhaustively evaluate all the neuropsychological aspects of MS. In order to obtain a more detailed view of patient’s cognition and perspective, self-evaluation questionnaires, mainly focused on perception of cognitive difficulties, fatigue and depression, are used in clinical practice. Namely, (i) fatigue, the so-called “invisible symptom” that interferes with cognitive, psychological and physical health, is commonly evaluated in MS patients by means of Fatigue Severity Scale (Nunnari et al., 2015; Ayache and Chalah, 2017; Lorefice et al., 2018); (ii) depression is evaluated with the Beck Depression Inventory-Second Edition (Siegert and Abernethy, 2005; Morrow et al., 2016; Patel and Feinstein, 2018); (iii) the Multiple Sclerosis Neuropsychological Questionnaire (MSNQ), a specific questionnaire that evaluates the perception of cognitive difficulties, is usually administrated, considering the frequent discrepancies between an objective assessment and the subjective viewpoint (Benedict et al., 2002; Julian et al., 2007; Kinsinger et al., 2010).

Taking into account all the above considerations, we tested to what extent D-KEFS ST could bring to light additional aspects of the cognitive impairment in MS not revealed by BRB-NT, but with potential relevant impact on patient’s daily life.

## Materials and Methods

### Patients

Hundred thirty-seven Relapsing-Remitting MS patients were enrolled in the study. At inclusion, the MS cohort had a mean age of 38.5 ± 10.9 years (range: 18–68), a mean disease duration of 8.4 ± 9.5 years (range: 0–42), a mean annualized relapse rate of 0.7 ± 0.5 (range: 0.0–2.0) and a median EDSS of 2.5 (range 0.0–7.0).

All the patients referred to the Multiple Sclerosis Centre of University Hospital of Padova for diagnosis (Polman et al., 2011) or clinical follow-up between March 2015 and April 2016. Exclusion criteria were history of other medical illness, learning disability, alcohol or drug abuse, impaired vision or hearing, and major psychiatric disorders.

The study was approved by the Ethic Committee of the Hospital of Padova. All the patients gave their written informed consent.

### Neuropsychological and Neurological Evaluations

The neuropsychological evaluation consisted in the administration during the same assessment of BRB-NT and D-KEFS ST, in agreement with the original manuals and normative data for Italian population (Amato et al., 2006a; Goretti et al., 2014; Mattioli et al., 2014). BRB-NT included the Selective Reminding Test (Long Term Storage-LTS; Consistent Long Term Retrieval-CLTR; SRT-D), the Spatial Recall Test (SPART, SPART-D), the Symbol Digit Modalities Test (SDMT), the Paced Auditory Serial Addition Test (PASAT) and the Word List Generation (WLG). D-KEFS ST comprehended the Free Sorting Description (D-KEFS ST FSD) the Free Sorting Categorization (D-KEFS ST FSC), and the Sort Recognition (D-KEFS ST SR). Corrected values (for age, gender and education) and z-scores were considered in the analysis. The failure in one item was defined when the z-score was below 1.5 standard deviation.

Self-evaluation questionnaires, i.e., the Multiple Sclerosis Neuropsychological Questionnaire (MSNQ; Benedict et al., 2003) the Fatigue Severity Scale (FSS; Krupp et al., 1989) and the Beck Depression Inventory II (BDI-II; Beck et al., 1996) were also completed by all the patients.

The administration of the whole neuropsychological assessment took around 60 min.

The neurological examination was performed by trained neurologists (FR, MP, PP, and PG), specifying the EDSS score.

### Statistics

ANOVA with Bonferroni’s correction was performed in order to evaluate normally distributed variables within the three patient’s subgroups identified (i.e., BRB-NT+D-KEFS ST cognitive impaired; BRB-NT impaired; cognitive normal). For qualitative ordinal variables the Mann-Whitney *U*-test was performed, while for qualitative nominal variables, the Pearson’s Chi square test was used. When all variables were normally distributed, correlations between variables were tested using the Pearson’s single or multiple linear models, while for ordinal variables Spearman correlation was applied. Finally, for dichotomic variables logistic regression analysis was also applied considering the dichotomic as dependent variable. The significance level was set at *p* < 0.05.

## Results

### BRB-NT+D-KEFS ST Impaired Patients Had Longer Disease Duration and Higher EDSS

Fifty-four patients (39.4%) had normal scores in each item of both batteries (cognitive normal), while 64 patients (46.7%) failed in at least one test of BRB-NT but not of D-KEFS ST (BRB-NT impaired) and 18 (13.1%) failed in at least one test of both batteries (BRB-NT+D-KEFS ST impaired). Only one patient (0.7%) failed in D-KEFS ST, but not in BRB-NT and was excluded from further analysis. Table 1 shows the main clinical and demographic variables of these groups. Cognitive normal patients were younger than BRB-NT impaired (*p* < 0.05) and BRB-NT+D-KEFS ST impaired patients (*p* < 0.005). BRB-NT+D-KEFS ST impaired patients had a longer disease duration compared to both cognitive normal (*p* < 0.005) and BRB-NT impaired patients (*p* < 0.01). Moreover, BRB-NT+D-KEFS ST impaired patients had a significant higher median EDSS score (3.5) compared to both BRB-NT impaired (2.5, *p* < 0.05) and cognitive normal patients (2.0, *p* < 0.01).

**Table 1:** Study population: clinical and demographic features.

	Overall cohort (137 patients)	Cognitive normal (54 patients)	BRB-NT impaired (64 patients)	BRB-NT+ D-KEFS ST impaired (18 patients)
Age	38.5 ± 10.9 (18–68)	34.7 ± 9.9 (18–56)	39.9 ± 11.0^∗^ (18–68)	45.0 ± 9.6^∗∗∗^ (21–60)
Gender (F/M ratio)	1.9	2.2	2.6	1.7
EDSS	2.5 (0.0–7.0)	1.8 (0.0–7.0)	2.5 (0.0–6.5)	4.0 (1.5–6.5)
Disease duration (years)	8.4 ± 9.5 (0.1–42.1)	6.5 ± 6.7 (0.3–32.9)	8.0 ± 9.3^§§^ (0.1–36.1)	15.5 ± 13.6^∗∗∗^ (0.6–42.1)
Education (years)	12.8 ± 3.7 (5–21)	12.6 ± 3.5 (8–20)	13.2 ± 3.7 (5–21)	11.9 ± 3.9 (8–18)


*Age and disease duration are calculated at neuropsychological evaluation. Cognitive normal, i.e., normal scores in each item of both batteries; BRB-NT impaired; i.e., patients failing in at least one test of BRB-NT but not of D-KEFS ST; BRB-NT+D-KEFS ST impaired; i.e., patients failing in at least one test of both batteries. Comparison with Cognitive Normal: ^∗^p < 0.05 and ^∗∗∗^p < 0.005. Comparison with BRB-NT+D-KEFS ST Impaired: ^§§^ p < 0.01.*

### D-KEFS ST Is Predicted by BRB-NT Failure

**FIGURE 1:**
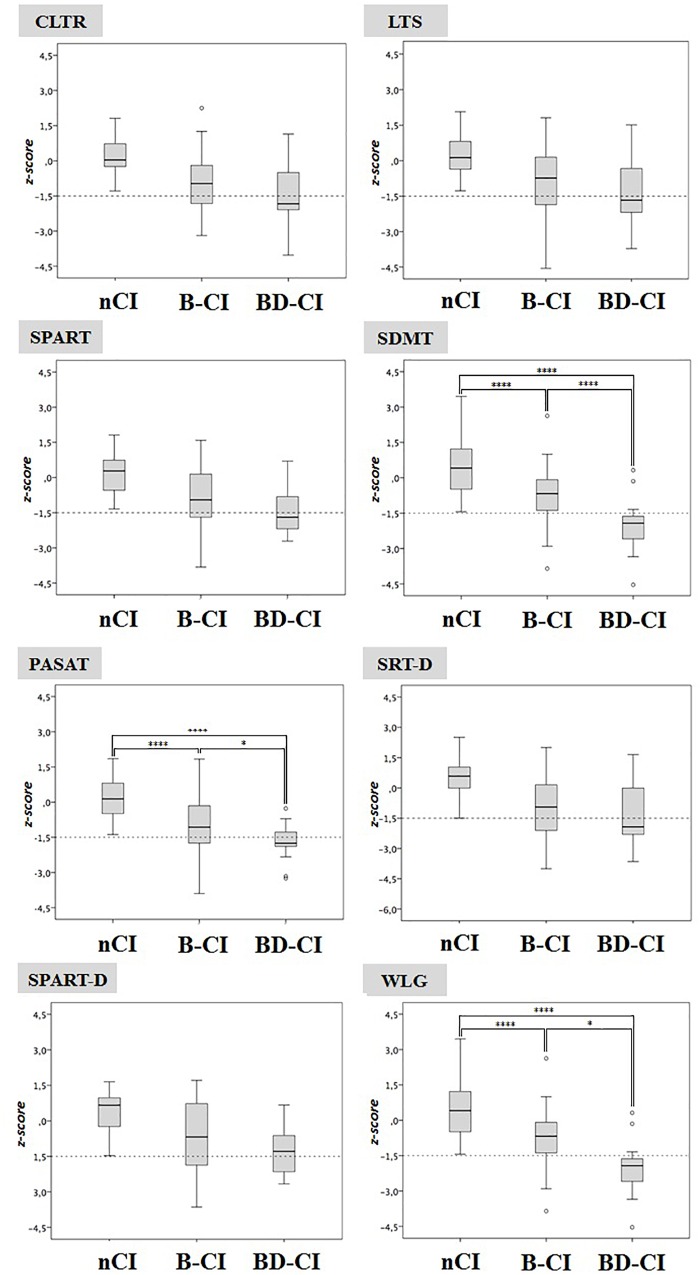
Cognitive normal, BRB-NT impaired and BRB-NT+D-KEFS ST impaired z-scores in BRB-NT items. While no difference was observed between cognitive normal and BRB-NT impaired, BRB-NT+D-KEFS ST impaired had significant lower SDMT and WLG *z*-values. ^∗^*p* < 0.05, ^∗∗^*p* < 0.01, ^∗∗∗^*p* < 0.005, and ^∗∗∗∗^*p* < 0.001.

BRB-NT+D-KEFS ST impaired patients had significant lower SDMT, PASAT and WLG *z*-values (-2.0 ± 1.1, -1.7 ± 0.8, and -0.9 ± 1.0, respectively) compared to BRB-NT impaired (-0.7 ± 1.1, *p* < 0.001, -0.9 ± 1.3, *p* < 0.05, and -0.2 ± 1.2 *p* < 0.05) and cognitive normal patients (0.4 ± 1.2, *p* < 0.001, 0.0 ± 1.0, *p* < 0.001, and -0.3 ± 1.0, *p* < 0.001) (Figure 1). SDMT, but not WLG or PASAT, was also more frequently impaired in BRB-NT+D-KEFS ST impaired patients (77.8%) than in BRB-NT impaired ones (18.8%, *p* < 0.001). All values and comparisons are in Supplementary Materials.

Logistic regression analysis disclosed that the failure in D-KEFS ST (i.e., pathological z-score in at least one out of three subcomponents) was strongly associated with the number of failed BRB-NT items (OR 1.46, IC_95%_ 1.07–1.99, *p* < 0.05) and with pathological SDMT *z*-values (OR 10.56, IC_95%_ 2.50–44.66, *p* < 0.005). No association with other variables (especially, EDSS, disease duration and age) was disclosed. These findings were confirmed for each D-KEFS ST subtest (Table 2).

**Table 2:** The impairment of D-KEFS ST Free Sorting Categorization (FSC), D-KEFS ST Free Sorting Description (FSD), and D-KEFS ST Sort Recognition (SR) associates with BRB-NT.

	Variables	OR	IC_95%_	*p*-value
D-KEFS ST FSC	*n° of failed BRB-NT items*	1.51	1.06–2.15	<0.05
	*Pathological SDMT*	9.91	1.57–62.64	<0.05
D-KEFS ST FSD	*n° of failed BRB-NT items*	1.91	1.21–3.00	<0.01
	*Pathological SDMT*	10.32	1.00–107.53	<0.05
D-KEFS ST SR	*n° of failed BRB-NT items*	1.51	1.08–2.10	<0.05
	*Pathological SDMT*	13.43	2.85–63.23	<0.005


*Regression analysis confirmed that impaired D-KEFS ST subtests correlated with SDMT and the number of failed BRB-NT items.*

### The Failure in D-KEFS ST Associates With Worse MSNQ and BDI-II Values

**FIGURE 2:**
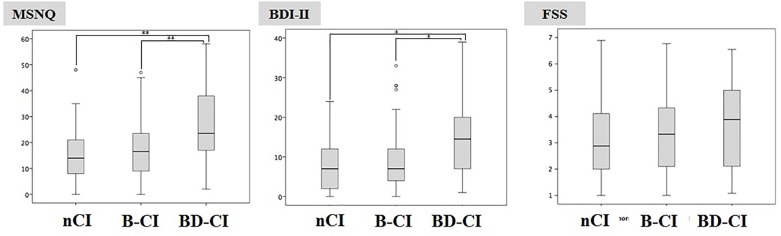
Multiple Sclerosis Neuropsychological Questionnaire and BDI-II values in Cognitive normal, BRB-NT impaired and BRB-NT+D-KEFS ST impaired. While no difference was observed between Cognitive Normal and BRB-NT impaired, BRB-NT+D-KEFS ST impaired had significant higher MSNQ and BDI-II.

BRB-NT+D-KEFS ST impaired patients had significant higher MSNQ and BDI-II values (26.7 ± 14.4 and 14.4 ± 9.3, respectively) compared to BRB-NT impaired (17.3 ± 11.1, *p* < 0.01 and 9.3 ± 7.8, *p* < 0.05) and cognitive normal patients (16.1 ± 10.9, *p* < 0.01 and 8.3 ± 6.8, *p* < 0.05) (Figure 2). All values and comparisons are in Supplementary Materials.

## Discussion

We found that all MS patients failing D-KEFS ST also failed BRB-NT, indicating that BRB-NT is more sensitive than D-KEFS ST in recognize cognitive impairment in MS. Moreover, SDMT, one of the most sensitive tests to identify early cognitive decline in MS patients, was more frequently impaired and associated with executive dysfunctions observed by D-KEFS ST. In addition, as disclosed by logistic regression analysis, the number of failed BRB-NT items and the presence of impaired SDMT strongly associated with higher probability of a pathological D-KEFS ST z-score. Interestingly, WLG, the only test of BRB-NT that theoretically could explore a component of executive functions (namely, the categorization process) did not associate with D-KEFS ST. Taken all together, our data suggest that patients failing in SDMT have the highest risk of failure in executive functions and require a more detailed analysis of cognition, which should include D-KEFS ST.

On the other hand, the self-reported questionnaires analysis clearly indicated that depression and high perception of cognitive failures characterize exclusively MS patients failing D-KEFS ST. Although self-evaluation questionnaires are not considered an objective assessment, this finding might help clinicians to better understand the impact of disability on patient’s quality of life. Furthermore, since D-KEFS ST specifically identifies patients having more severe clinical (i.e., higher EDSS score) and neuropsychological impairments, this test may be useful to stratify MS subgroups for clinical studies.

A further relevant clinical rebound of our observations concerns the therapeutic management of the patients. It has been observed that the integrity of executive functions is associated with a better therapeutic compliance (Brock et al., 2011) and this aspect may be relevant in MS patients that manage drugs whose administration must be carefully scheduled, imply appropriate and specific clinical and laboratory follow-up and may present adverse events that must be quickly reported to clinicians. Thus, the presence of executive dysfunctions may significantly influence therapeutic decision-making (i.e., in favor of simpler therapeutic protocols) and should be explored in all MS patients that are initiating disease-modifying drugs. Whether D-KEFS ST may be used to predict therapeutic failures due to lack of adherence merits to be evaluated.

Finally, we are aware that the lack of a normal reference group might be considered a limit of our work. Nevertheless, we applied z-score analysis (validated for Italian population) (Amato et al., 2006a; Goretti et al., 2014; Mattioli et al., 2014) to weight correctly the observed differences.

## Conclusion

In conclusion, although D-KEFS ST did not increase the number of cognitively impaired MS patients identified by BRB-NT, it provided a more comprehensive evaluation of the cognitive decline. In particular, D-KEFS ST failure identified a subgroup of patients with increased self-perception of cognitive dysfunctions, depression and higher physical disability. Whether these patients may be therapy-failures for lack of adherence merits further investigation in longitudinal studies.

## Ethics Statement

The study was approved by the Local Ethic Committee. All the patients gave their informed consent.

## Author Contributions

AR designed the study and collected the neuropsychological data. MP designed the study and performed the data analysis. FR and PP collected clinical data. ME performed data analysis. PG designed the study and contributed to the final version of the manuscript.

## Conflict of Interest Statement

AR reports grants and personal fees from Novartis, grants and personal fees from Biogen Idec, grants from Teva, grants from Merck Serono, during the conduct of the study. MP reports grants and personal fees from Almirall, grants from Teva, grants and personal fees from Genzyme Sanofi, grants from Merck Serono, grants and personal fees from Biogen Idec, grants and personal fees from Novartis, during the conduct of the study. FR reports grants from Almirall, grants and personal fees from Teva, grants and personal fees from Genzyme Sanofi, grants and personal fees from Merck Serono, grants and personal fees from Biogen Idec, grants from Novartis, during the conduct of the study. ME has nothing to disclose. PP reports grants and personal fees from Merck Serono, grants and personal fees from Biogen Idec, grants and personal fees from Genzyme Sanofi, grants and personal fees from Bayer Schering Pharma, grants and personal fees from Novartis, grants and personal fees from Teva, during the conduct of the study. PG reports grants and personal fees from Merck Serono, grants and personal fees from Biogen Idec, grants and personal fees from Genzyme Sanofi, grants and personal fees from Bayer Schering Pharma, grants and personal fees from Novartis, grants and personal fees from Teva, grants from University of Padua, Department of Neurosciences DNS, grants from Veneto Region of Italy, grants from Italian Association for Multiple Sclerosis (AISM), grants from Italian Ministry of Public Health, during the conduct of the study.
