# Supplementary material for: D-KEFS ST Failure Identifies Multiple Sclerosis Patients With Worse Objective and Self-Perceived Physical and Cognitive Disability
Source: Front Psychol. 2019 Jan 24;10:49. doi: 10.3389/fpsyg.2019.00049 (PMC6353833; doi:10.3389/fpsyg.2019.00049)
Supplement: Supplementary file 1 [file Data_Sheet_1.docx]

***Supplementary Materials.***

***Neuropsychological data.*** For each test data are expressed as z-score and as percentage of patients with an impaired z-score. Abbreviations: Cognitive Normal, i.e. normal scores in each item of both batteries; BRB-NT Impaired; i.e. patients failing in at least one test of BRB-NT but not of D-KEFS ST; BRB-NT+D-KEFS ST Impaired, i,e. patients failing in at least one test of both batteries; LTS: Long Term Storage; CLTR: Consistent Long-Term Retrieval; SRT-D: Selective Reminding Test D; SPART and SPART-D: Spatial Recall Test; SDMT: Symbol Digit Modalities Test; PASAT: Paced Auditory Serial Addition Test; WLG: Word List Generation; D-KEFS ST FSD: Delis-Kaplan Executive Function System Free Sorting Description; D-KEFS FSC: Delis-Kaplan Executive Function System Free Sorting Categorization; D-KEFS ST SR: Delis-Kaplan Executive Function System Sort Recognition. Comparison with BRB-NT+D-KEFS Impaired: *: p<0.05; **: p<0.01; ***: p<0.005; ****<0.001; comparison with BRB-NT Impaired: ^§^: p<0.05; ^§§§§^:p<0.001.

|  | **Cognitive Normal (54 patients)** | **BRB-NT Impaired (64 patients)** | **BRB-NT+**  **D-KEFS ST Impaired (18 patients)** |
| --- | --- | --- | --- |
| LTS (z-score) | 0.14±0.88****^§§§§^ | 0.80±1.35 | -1.44±1.47 |
| CLTR (z-score) | 0.11±0.85****^§§§§^ | -0.94±1.16 | -1.50±1.20 |
| SPART (z-score) | 0.08±0.82****^§§§§^ | -0.77±1.26 | -1.34±1.02 |
| SDMT (z-score) | 0.40±1.20****^§§§§^ | -0.73±1.05**** | -2.04±1.09 |
| PASAT (z-score) | 0.00±1.01****^§§§§^ | -0.88±1.27* | -1.70±0.82 |
| SRT-D (z-score) | 0.39±0.99****^§§§§^ | -0.99±1.52 | -1.41±1.54 |
| SPART-D (z-score) | 0.35±0.78****^§§§§^ | -0.60±1.41 | -1.28±1.04 |
| WLG (z-score) | 0.27±0.98****^§§§§^ | -0.21±1.21* | -0.92±1.01 |
| S-KEFS ST FSC (z-score) | -0.23±0.67****^§^ | -0.55±0.50 | -2.02±1.00 |
| S-KEFS ST FSD (z-score) | 0.05±0.62****^§^ | -0.26±0.47 | -1.71±0.91 |
| S-KEFS ST SR (z-score) | -0.08±0.54****^§§^ | -0.39±0.56 | -2.34±0.92 |
| MSNQ (z-score) | 15.8±11.1*** | 17.5±10.9** | 26.8±14.4 |
| FSS (z-score) | 3.1±1.5 | 3.4±1.4 | 3.8±1.8 |
| BDI-II (z-score) | 8.1±6.8** | 9.5±7.8* | 14-4±9.3 |
| LTS (%) | 1.9% | 28.1%* | 61.1% |
| CLTR (%) | 1.9% | 32.8% | 61.1% |
| SPART (%) | 0% | 34.4% | 55.6% |
| SDMT (%) | 1.9% | 18.8%**** | 77.8% |
| PASAT (%) | 3.7% | 39.1% | 55.6% |
| SRT-D (%) | 3.7% | 40.6% | 55.6% |
| SPART-D (%) | 0% | 32.8% | 44.4% |
| WLG (%) | 1.9% | 15.6% | 33.3% |
| S-KEFS ST FSC (%) | 0% | 0% | 66.7% |
| S-KEFS ST FSD (%) | 0% | 0% | 61.1% |
| S-KEFS ST SR (%) | 0% | 0% | 94.4% |
